# Geometric morphometrics and machine learning from three-dimensional facial scans for difficult mask ventilation prediction

**DOI:** 10.3389/fmed.2023.1203023

**Published:** 2023-08-10

**Authors:** Bei Pei, Chenyu Jin, Shuang Cao, Ningning Ji, Ming Xia, Hong Jiang

**Affiliations:** Department of Anaesthesiology, Shanghai Ninth People’s Hospital, Shanghai Jiao Tong University School of Medicine, Shanghai, China

**Keywords:** difficult airway, difficult mask ventilation, predictive model, machine learning, three dimension scanning, geometric morphometrics

## Abstract

**Background:**

Unanticipated difficult mask ventilation (DMV) is a potentially life-threatening event in anesthesia. Nevertheless, predicting DMV currently remains a challenge. This study aimed to verify whether three dimensional (3D) facial scans could predict DMV in patients scheduled for general anesthesia.

**Methods:**

The 3D facial scans were taken on 669 adult patients scheduled for elective surgery under general anesthesia. Clinical variables currently used as predictors of DMV were also collected. The DMV was defined as the inability to provide adequate and stable ventilation. Spatially dense landmarks were digitized on 3D scans to describe sufficient details for facial features and then processed by 3D geometric morphometrics. Ten different machine learning (ML) algorithms, varying from simple to more advanced, were introduced. The performance of ML models for DMV prediction was compared with that of the DIFFMASK score. The area under the receiver operating characteristic curves (AUC) with its 95% confidence interval (95% CI) as well as the specificity and sensitivity were used to evaluate the predictive value of the model.

**Results:**

The incidence of DMV was 35/669 (5.23%). The logistic regression (LR) model performed best among the 10 ML models. The AUC of the LR model was 0.825 (95% CI, 0.765–0.885). The sensitivity and specificity of the model were 0.829 (95% CI, 0.629–0.914) and 0.733 (95% CI, 0.532–0.819), respectively. The LR model demonstrated better predictive performance than the DIFFMASK score, which obtained an AUC of 0.785 (95% CI, 0.710–0.860) and a sensitivity of 0.686 (95% CI, 0.578–0.847). Notably, we identified a significant morphological difference in the mandibular region between the DMV group and the easy mask ventilation group.

**Conclusion:**

Our study indicated a distinct morphological difference in the mandibular region between the DMV group and the easy mask ventilation group. 3D geometric morphometrics with ML could be a rapid, efficient, and non-invasive tool for DMV prediction to improve anesthesia safety.

## 1. Introduction

Airway management is a critical aspect of ensuring the safety and quality of anesthesia. Mask ventilation (MV) is a cornerstone of airway management, serving as both an initial ventilation technique and a rescue method during difficult or failed tracheal intubation (1). Difficult mask ventilation (DMV) was reported to be an essential factor for severe airway-related complications such as death or hypoxic brain injury in anesthesia (2). As a result, it is essential to conduct a thorough assessment of the patient’s airway before the induction of anesthesia. For patients with a high risk of DMV, the anesthesiologists can prepare alternative approaches in advance such as a plan for awake fiberoptic intubation to ensure safety (3).

Abnormal facial features can directly impact external mask fit, which potentially makes mask ventilation more challenging, and thus, the patient’s morphology may be a relevant predictor for DMV. Recently, two-dimensional (2D) images and three-dimensional (3D) scans have been employed to characterize the maxillofacial structure and predict diseases (4, 5). In the field of anesthesia, 2D images have been implemented to construct a predictive model for the classification of difficult intubation (6, 7). However, 2D images are susceptible to external factors such as lighting, which may affect their accuracy. Moreover, human faces are inherently 3D objects, and 2D images are merely projections of the face on a flat surface, thus potentially resulting in a loss of important characteristics. To address these limitations, 3D scans are more suitable for examining the complex structures of facial shapes with greater reliability.

Conventional morphometric analysis that relies on linear measurements such as angles or lengths may not capture the complex variation in 3D shapes. Geometric morphometrics is a more effective tool as it can retain geometric information such as the relative position of each structure, allowing for quantification and visualization of morphometric results (8). For instance, the recent development in 3D craniofacial scans and geometric morphometric analysis has shown promising results in predicting obstructive sleep apnea (OSA), surpassing the performance of traditional questionnaires (9). It has been verified that there is a relationship between DMV and OSAS (10), and they share common morphological features, such as retrognathia and a thick neck.

No study has explored the relationship between 3D facial scans and DMV to our knowledge, so here we proposed that 3D geometric morphometric analysis of facial scans combined with machine learning (ML) algorithms could be an alternative tool to predict DMV in patients scheduled for general anesthesia.

## 2. Materials and methods

### 2.1. Patients

This observational study was conducted between June 2021 and January 2022 after obtaining approval from the Ethics Committee of Shanghai Ninth People’s Hospital (no. SH9H-2020-T233-1). The protocol is registered on ClinicalTrials.gov (trial registration no. NCT 04458220). The study was conducted in accordance with the Declaration of Helsinki (as revised in 2013).

The inclusion criteria for the study were adult patients scheduled for elective surgery under general anesthesia. The exclusion criteria were as follows: with mental or central nervous system disease; with stupefaction or disturbance of consciousness; with terrible injury; with difficulties in communicating; cannot follow instructions to make standardized postures; participated in other relevant clinical investigation in the past 3 months. Informed consent was provided by each participant before their inclusion.

### 2.2. Preoperative airway assessment

The demographic properties of patients’ age, gender, weight, height, and body mass index (BMI) were collected during the preoperative visit. Drawing inspiration from a previous study that developed a weighted risk score for DMV prediction named DIFFMASK score (11), we collected additional data including the history of snoring, history of obstructive sleep apnea, history of neck radiation, history of difficult tracheal intubation, modified Mallampati test (MMT), and thyromental distance (TMD).

All researchers received repeated training before this trial to reduce measurement bias. The modified Mallampati test (MMT) was conducted with patients in full neck extension, while being asked to open their mouths widely and protrude their tongues, without vocalizing (12). The thyromental distance was determined by measuring the distance between the uppermost border of the thyroid cartilage and the mentum, with the neck in an extended position (12).

### 2.3. 3D geometric morphometrics of the craniofacial structure

#### 2.3.1. Facial surface imaging

All 3D scans were acquired in the Shanghai Ninth People’s Hospital by the same researcher who was specifically trained prior to the trial to ensure the uniformity of data.

A 3D face scanner, FaceGo pro (Revopoint, China) was utilized to generate 3D facial models with an accuracy of 0.1 mm. Participants were instructed to fully expose their face and neck region, maintain a neutral facial expression, and look parallelly at the camera during the scanning process, with their heads in a natural position. Each participant was asked to keep the head still during the whole scan which could be finished in 1 min.

#### 2.3.2. Manual annotation

**Figure 1:**
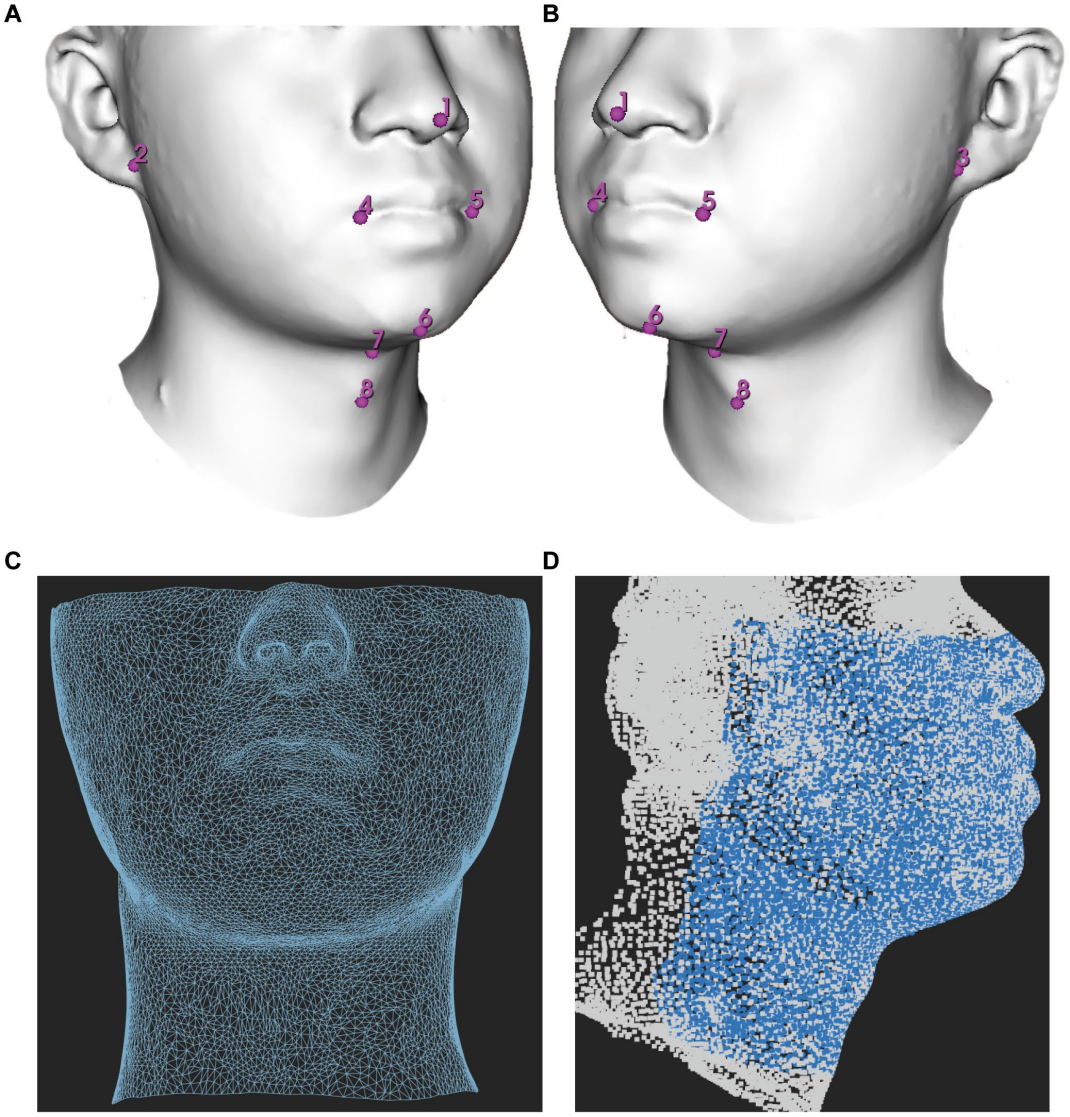
Demonstration of facial mapping. **(A)** Digitization of eight anchoring points on a 3D model in a right lateral view. **(B)** Digitization of eight anchoring points on a 3D model in a right left view. **(C)** The reference mesh, consisting of 9,578 vertices and 18,812 faces formed by three adjacent vertices, was illustrated as a wireframe model. **(D)** Spatially dense facial landmarks (blue) were mapped onto reference mesh shown in a lateral view illustrated as a point cloud model.

The models were saved in OBJ format and subsequently processed using Meshmixer (release 3.5.474)^1^ to eliminate the redundant parts. Each facial scan in OBJ format was imported into the 3D Slicer (release 5.0.3)^2^ which is an open-source biomedical visualization and image analysis software supported by the National Institutes of Health (NIH) (13) to digitize 8 anchoring points (pronasale, right earlobe, left earlobe, right cheilion, left cheilion, tip of the chin, hyoid bone, and thyroid notch) in a fixed order (Figures 1A,B). The placement of anchoring points was performed by a single researcher to minimize potential user bias.

#### 2.3.3. Spatially dense surface registration

All acquisitions were mapped using MeshMonk, an open-source software toolbox available at https://github.com/TheWebMonks/meshmonk, within MATLAB 2018b. MeshMonk facilitates spatially dense registration of 3D surfaces (14). Through iterative rigid and non-rigid registration algorithms, MeshMonk enables the alignment of each 3D surface to a reference mesh.

A single patient with a fully exposed head and neck region and minimal caveats was selected as the reference mesh. The choice of reference mesh has little impact on statistics, as long as it fulfills the criteria of having no significant holes and uniform vertex coverage (15).

The reference mesh was subsequently cleaned and prepared using Meshmixer (version 3.5.474), accessible at https://meshmixer.com/. The cleanup process aimed to retain the area below the eyes and above the plane of the thyroid cartilage, as it held significant interest for DMV shown in Figure 1C. Our hypothesis was that this region, from below the eyes to above the jaw, could affect mask ventilation by influencing mask fit while the region of mandible and neck could potentially interfere with mask ventilation by impacting airflow. Following the cleanup, the reference mesh consisted of 9,578 vertices. The reference mesh in OBJ format could be found in Supplementary file.

Subsequently, the reference mesh underwent iterative rigid and non-rigid registration algorithms to align each facial image. As the same reference mesh was used, the landmarks redefined on each facial sample were matched point-to-point consistently across all samples (16).

To explore the potential impact of using different reference meshes from different patients, we randomly selected three additional patients. Subsequently, each facial image was aligned to different reference mesh for subsequent analyses.

#### 2.3.4. Generalized procrustes analysis

A Generalized Procrustes analysis (GPA) was then applied to re-align all meshes into a common coordinate system, using a total of 9,578 quasi-landmarks which removed among configuration variations in size, location, and orientation (17).

### 2.4. Dimensionality reduction

A total of 9,578 quasi-landmarks were available to characterize each patient’s maxillofacial and neck shape. A principal component analysis (PCA) was then applied to the Procrustes-aligned coordinates to reduce the dimensionality of the data and extract a smaller set of orthogonal dimensions that captured the variability in the dataset. A linear discriminant analysis (LDA) was employed using a simple Leave-One-Out Cross-Validation (LOOCV) technique systematically increasing the number of principal components (PCs) from 1 to 50 as input to determine the optimal number of PCs for predicting DMV. In LOOCV, one sample was used as the validation data, while the rest were used as the training data. This process was repeated such that each sample in the dataset was used once as the validation data. The optimal number of PCs for predicting DMV was determined based on the highest value of the area under the receiver operating characteristic curve (AUC).

The morphometric data was processed by the R project software program (R 4.2.2)^5^ mainly using geomorph (18) and Morpho packages (19). The LDA used MASS packages and the self-generated code was developed to implement LOOCV.

### 2.5. Induction of anesthesia and MV evaluation

Airway management was conducted by an anesthesiologist with over 3 years of experience. General anesthesia was induced with a combination of midazolam 0.05 mg/kg, fentanyl 2–4 μg/kg, propofol 2–2.5 mg/kg, and rocuronium 0.6 mg/kg. The patient’s head was placed in the ‘sniffing position’ by extending the neck and throughout the procedure, electrocardiography, noninvasive blood pressure, end-tidal carbon dioxide, and peripheral oxygen saturation (SpO_2_) were continuously monitored.

During the induction of anesthesia, the anesthesiologist was instructed to employ a one-handed technique for airway opening. This involved holding the anesthesia full-face mask (Flexicare, United Kingdom; sizes 3 and 4) with their thumb and index fingers while positioning the third and fourth fingers on the left mandibular ramus, and placing the fifth finger at the left mandibular angle.

Following the induction of anesthesia, pressure-controlled ventilation was initiated through the full-face mask *via* an anesthesia machine ventilator, with a peak inspiratory pressure of 15 cm H_2_O, positive end-expiratory pressure of 0, I: E ratio of 0.4, and a respiratory rate of 15 cycles per minute for a duration of 2 min.

During face mask ventilation, one-handed technique without adjuvant (such as oral airway and jaw thrust) by an unassisted anesthesiologist was routinely utilized. DMV was defined as the inability to achieve adequate ventilation using this technique. The inadequate ventilation was defined according to Langeron et al. (20) as follows: (1) the inability of an unassisted anesthesiologist to maintain oxygen saturation, as measured by SpO_2_ < 92% with 100% oxygen and positive-pressure mask ventilation; (2) important gas flow leakage around the face mask; (3) the need to increase the gas flow to more than 15 L/min and use the oxygen flush valve more than twice (4) absence of visible chest movement; (5) the necessity to switch to a two-handed mask ventilation technique; (6) the need for operator substitution.

In clinical practice, we observed that the perceptible chest movement was subjective so we also considered ventilation inadequate if the tidal volume was less than 5 mL/kg ideal body weight, following the study by Sato et al. (10).

To ensure the safety of patients if inadequate ventilation was encountered, steps were taken to address the situation effectively as recommended by the guidelines (21). This involved inserting an appropriately sized oral airway and applying an optimal jaw thrust technique while securely holding the mask with both hands. If these measures were unsuccessful, seeking help, changing the operator, or involving a two-person technique was considered. If adequate ventilation cannot be achieved, careful consideration is given to either waking patients using sugammadex to reverse the neuromuscular blockade induced by rocuronium or promptly establishing a noninvasive artificial airway, such as a supraglottic airway or endotracheal intubation. If these interventions also fail, cricothyrotomy should be performed immediately.

### 2.6. Machine learning algorithms

For the purpose of building a prediction model, a total of 10 ML algorithms, including Naive Bayes, linear discriminant analyses (LDA), quadratic discriminant analysis (QDA), logistic regression (LR), support vector machine (SVM), random forest (RF), extra trees, artificial neural network (ANN), adaptive boosting (AdaBoost), and extreme gradient boosting (XGBoost), representing diverse categories were performed using the morphometric data (22). Each algorithm has its own advantages and disadvantages, and our aim was to identify the most appropriate algorithm for our data. The model’s performance was assessed using the 10-fold cross-validation method (23). This approach involved dividing the cohort into ten folds. In each iteration of the cross-validation process, one fold was set aside for evaluation purposes, while the remaining nine folds were utilized for training the model. By iteratively changing the validation fold in each round of the cross-validation process, each part of the cohort served as the validation set exactly once. This process enhanced the robustness of the evaluation and contributed to a more reliable assessment of the model’s performance.

### 2.7. Statistical analysis

The measurement data were presented as mean ± standard deviation (SD), whereas categorical variables were expressed as frequency (%). The hypothesis was tested using one-way analysis of variance (ANOVA), the Mann–Whitney U test, and Fisher’s exact probability method. Statistical significance was defined as *p* < 0.05. To assess classification performance, the area under the receiver operating characteristic curve (AUC) with its 95% confidence interval (95% CI), as well as the sensitivity and specificity, were utilized as primary metrics. All data analysis was conducted utilizing the R project software program (R 4.2.2) (see footnote 3).

We used the method by Riley et al. to calculate for the efficient sample size (24). We did not calculate the sample size in advance because we utilized all accessible data throughout the study period. However, we did a *post hoc* sample size calculation to verify whether the developed models ensure accurate prediction. In our study, selecting an estimated C statistic of 0.825, a prevalence of DMV 5.23%, and a predictor parameters of 3, model development required at least 331 cases. Our total sample size included 669 patients which satisfied the minimum sample size requirement.

## 3. Results

### 3.1. Baseline characteristics

**Figure 2:**
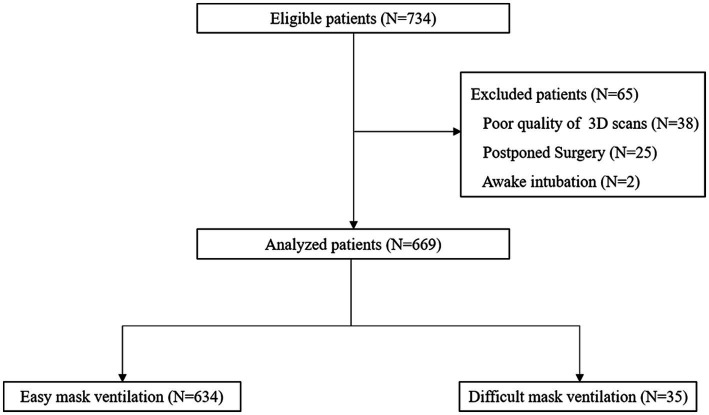
Flow chart of the study.

A total of 734 patients initially screened. Thirty-eight patients were excluded because of the poor quality of 3D scans. Twenty-five patients were excluded because of postponed surgery, and 2 patients were excluded because they underwent awake intubation. Finally, 669 patients were enrolled, including 634 patients with easy MV and 35 patients with DMV. A flow chart of the study is shown in Figure 2. The baseline characteristics of the study population are presented in Table 1. Statistical analysis revealed significant differences in age, gender, BMI, and snoring history between the DMV group and the easy MV group. Only a single patient in the DMV group had a history of neck radiation and difficult intubation. None of the patients received sugammadex or rescue ventilation devices.

**Table 1:** The baseline demographic properties and risk factors for patients included.

Risk factors	Overall (*n* = 669)	Easy MV (*n* = 634)	DMV (*n* = 35)	*p* value
Age (years)	34.67 ± 11.53	34.32 ±11.38	40.94 ±12.61	0.001
Gender				<0.001
Female	375 (56.1)	366 (57.7)	9 (25.7)	
Male	294 (43.9)	268 (42.3)	26 (74.3)	
BMI (kg/m^2^)	22.18 ± 3.41	21.90 ± 3.16	27.30 ± 3.64	<0.001
TMD (cm)	9.52 ± 1.35	9.50 ± 1.36	9.76 ± 1.13	0.269
Snoring history				<0.001
Yes	282 (42.2)	256 (40.4)	26 (74.3)	
No	387 (57.8)	378 (59.6)	9 (25.7)	
Neck radiation history				0.044
Yes	1 (0.1)	0 (0)	1 (2.9)	
No	668 (99.9)	634 (100.0)	34 (97.1)	
DI history				0.044
Yes	1 (0.1)	0 (0)	1 (2.9)	
No	668 (99.9)	634 (100.0)	34 (97.1)	
Sleep apnea				1.000
Yes	1 (0.1)	1 (0.2)	0 (0)	
No	668 (99.9)	633 (99.8)	35 (100.0)	
MMT				0.735
1	152 (22.7)	145 (22.9)	7 (20.0)	
2	223 (33.3)	212 (33.4)	11 (31.4)	
3	262 (39.2)	248 (39.1)	14 (40.0)	
4	32 (4.8)	29 (4.6)	3 (8.6)	

Data are shown as number (percentage) or mean ± SD; SD, standard deviation; MV, mask ventilation; DMV, difficult mask ventilation; BMI, body mass index; TMD, thyromental distance; DI, difficult intubation; MMT, modified Mallampati test.

### 3.2. The principal component analysis

Principal component analysis (PCA) demonstrated that the first three principal components (PCs) were responsible for describing 42.63% of the total variance in the data. 75% of the total variance can be described only by 14 PCs. The LDA was performed using a range of a range of PCs from 1 to 50 as input, with a LOOCV technique. The results showed that the highest AUC of 0.819 (95% CI, 0.758–0.880) was achieved when only the first 3 PCs were processed, with a sensitivity of 0.829 (95% CI, 0.657–0.943) and a specificity of 0.700 (95% CI, 0.513–0.765) when the highest point of the Youden index was the threshold.

**Figure 3:**
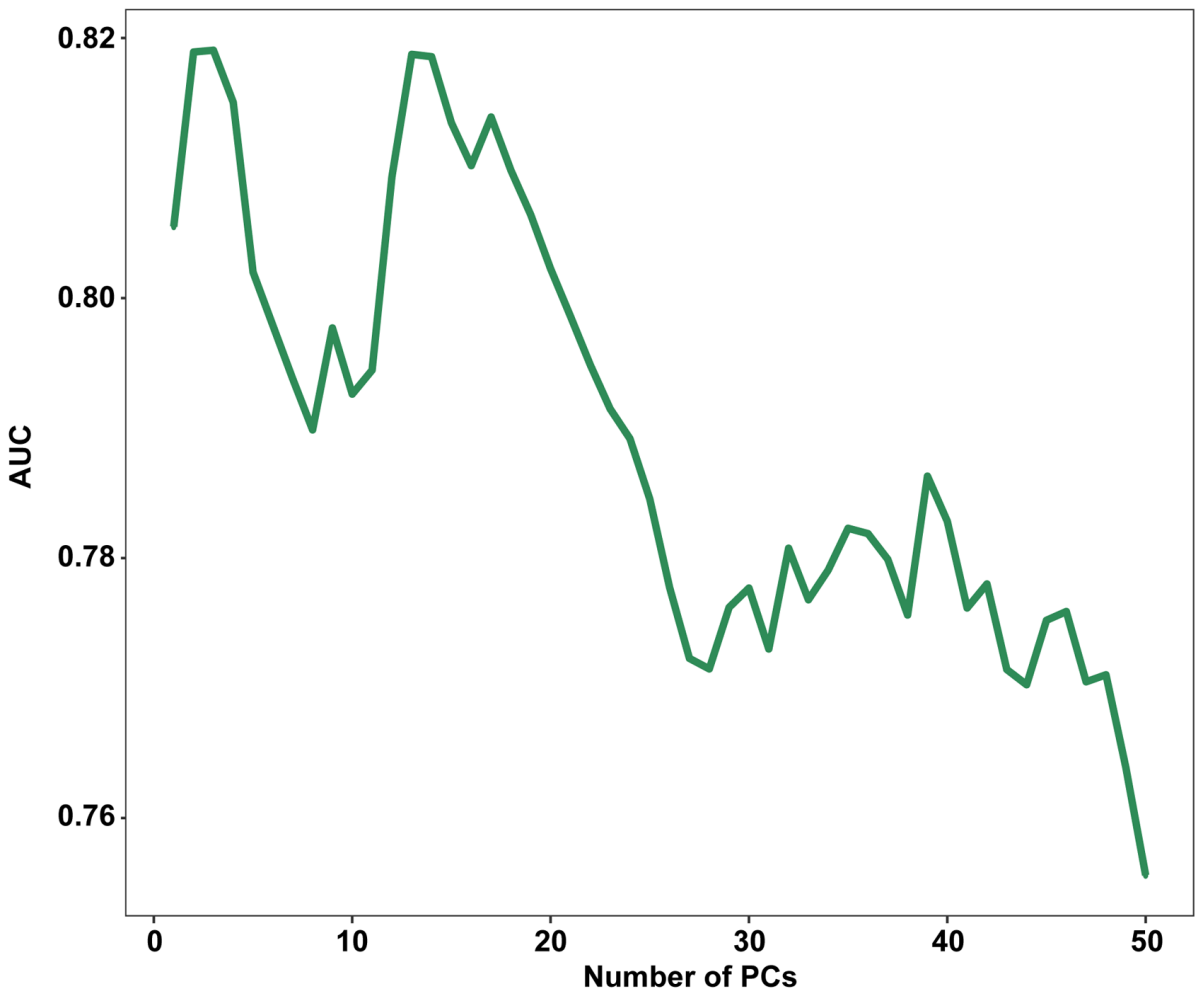
Influence of the number of PCs retained on the AUC score. PC, principal component; AUC, The area under the receiver operating characteristic curves.

After that, there was a brief decline in the performance of the model as the number of PCs increased, and then there was some improvement when with the first 14 PCs as input, but it still did not exceed the performance of using the first 3 PCs and after that the performance of the model continued decline as the number of PCs increased (Figure 3). This is the cost of dimensionality based on morphometric data in classification.

Using scans from 3 random participants as the reference mesh, realigned them with all patients’ scans, had a negligible effect on the performance of the models (Supplementary Table S1).

### 3.3. DMV prediction from morphometric data

**Figure 4:**
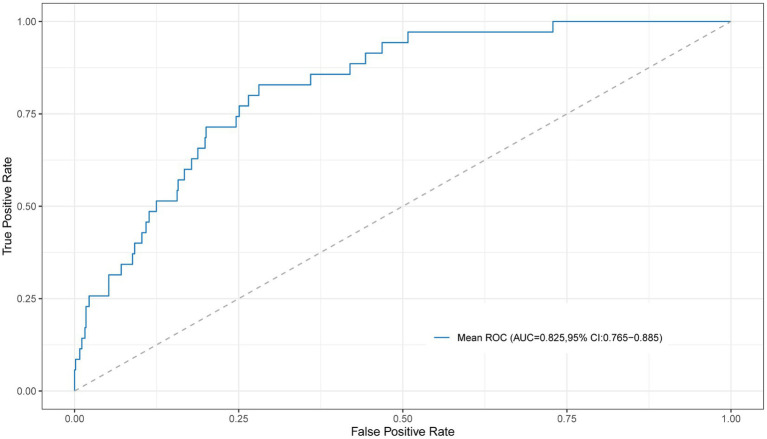
The ROC curve for the LR model with 10-fold cross-validation using the first 3 PCs as input. ROC, receiver operating characteristic; PC, principal component.

Based on the preliminary test results, we observed two peaks in the first 2 to 5 PCs and the first 13 to 15 PCs. Consequently, we chose to explore the first 2 to 5 PCs and 13 to first 13 to 15 PCs to further investigate the optimal number of PCs and identify the best algorithm for our analysis. The predictive performance was evaluated using the 10-fold cross-validation method (Table 2). The SVM, extra trees, and AdaBoost showed relatively poor performance. However, the other algorithms exhibited good predictive performance, with AUC over 0.80. At this step, the LR model was selected as the preferred algorithm due to its speed and superior performance. When only 3 PCs were input, this model achieved an AUC of 0.825 (95% CI, 0.765–0.885) by the 10-fold cross-validation method with a sensitivity of 0.829 (95% CI, 0.629–0.914), and a specificity of 0.733 (95% CI, 0.532–0.819) (Figure 4).

**Table 2:** The AUC (95% CI) of the models evaluated by 10-fold cross-validation using various machine learning algorithms with 2 to 5 PCs and 13 to 15 PCs inputs.

Variables	2 PCs (95% CI)	3 PCs (95% CI)	4 PCs (95% CI)	5 PCs (95% CI)	…	13 PCs (95% CI)	14 PCs (95% CI)	15 PCs (95% CI)
Naive Bayes	0.818 (0.754–0.881)	0.810 (0.746–0.872)	0.798 (0.731–0.865)	0.786 (0.713–0.860)	…	0.775 (0.702–0.847)	0.774 (0.702–0.846)	0.768 (0.696–0.841)
LDA	0.820 (0.759–0.882)	0.823 (0.764–0.882)	0.818 (0.758–0.877)	0.799 (0.728–0.870)	…	0.815 (0.746–0.884)	0.814 (0.746–0.882)	0.810 (0.740–0.881)
QDA	0.812 (0.747–0.878)	0.797 (0.728–0.867)	0.778 (0.707–0.850)	0.774 (0.698–0.851)	…	0.718 (0.627–0.809)	0.757 (0.672–0.843)	0.746 (0.665–0.826)
LR	0.823 (0.762–0.884)	0.825 (0.765–0.885)	0.820 (0.760–0.880)	0.805 (0.737–0.872)	…	0.818 (0.753–0.883)	0.818 (0.755–0.881)	0.815 (0.749–0.881)
SVM	0.560 (0.470–0.650)	0.608 (0.500–0.716)	0.569 (0.474–0.664)	0.546 (0.452–0.639)	…	0.706 (0.623–0.790)	0.645 (0.560–0.730)	0.678 (0.587–0.770)
RF	0.765 (0.683–0.846)	0.758 (0.678–0.837)	0.810 (0.733–0.887)	0.799 (0.717–0.881)	…	0.758 (0.667–0.849)	0.759 (0.664–0.855)	0.773 (0.685–0.862)
Extra trees	0.673 (0.579–0.766)	0.612 (0.514–0.711)	0.618 (0.521–0.715)	0.614 (0.521–0.708)	…	0.691 (0.602–0.781)	0.681 (0.587–0.774)	0.681 (0.587–0.774)
ANN	0.778 (0.700–0.856)	0.810 (0.741–0.877)	0.794 (0.720–0.868)	0.782 (0.701–0.862)	…	0.805 (0.732–0.878)	0.761 (0.684–0.839)	0.773 (0.695–0.852)
AdaBoost	0.726 (0.630–0.823)	0.686 (0.598–0.774)	0.759 (0.668–0.851)	0.761 (0.671–0.851)	…	0.761 (0.677–0.846)	0.725 (0.623–0.827)	0.732 (0.634–0.831)
XGBOOST	0.788 (0.700–0.876)	0.803 (0.725–0.881)	0.797 (0.718–0.875)	0.781 (0.694–0.868)	…	0.809 (0.732–0.886)	0.807 (0.729–0.885)	0.808 (0.730–0.886)

AUC, the area under the receiver operating characteristic curves; CI, confidence interval; PCs, principal components; LDA, linear discriminant analyses; QDA, quadratic discriminant analysis; LR, logistic regression; SVM, support vector machine; RF, random forest; ANN, artificial neural network; AdaBoost, adaptative boosting; XGBOOST, eXtreme Gradient Boosting.

### 3.4. Comparison to DIFFMASK score

The DIFFMASK score got an AUC of 0.785 (95% CI, 0.710–0.860). The Youden index identified a score ≥ 4 as the optimal cut-off value for DMV prediction, with a sensitivity of 0.686 (95% CI, 0.578–0.847) and a specificity of 0.785 (95% CI, 0.589–0.848). The performance of the morphometric data surpassed those of the DIFFMASK scores.

### 3.5. Visual prediction of DMV

**Figure 5:**
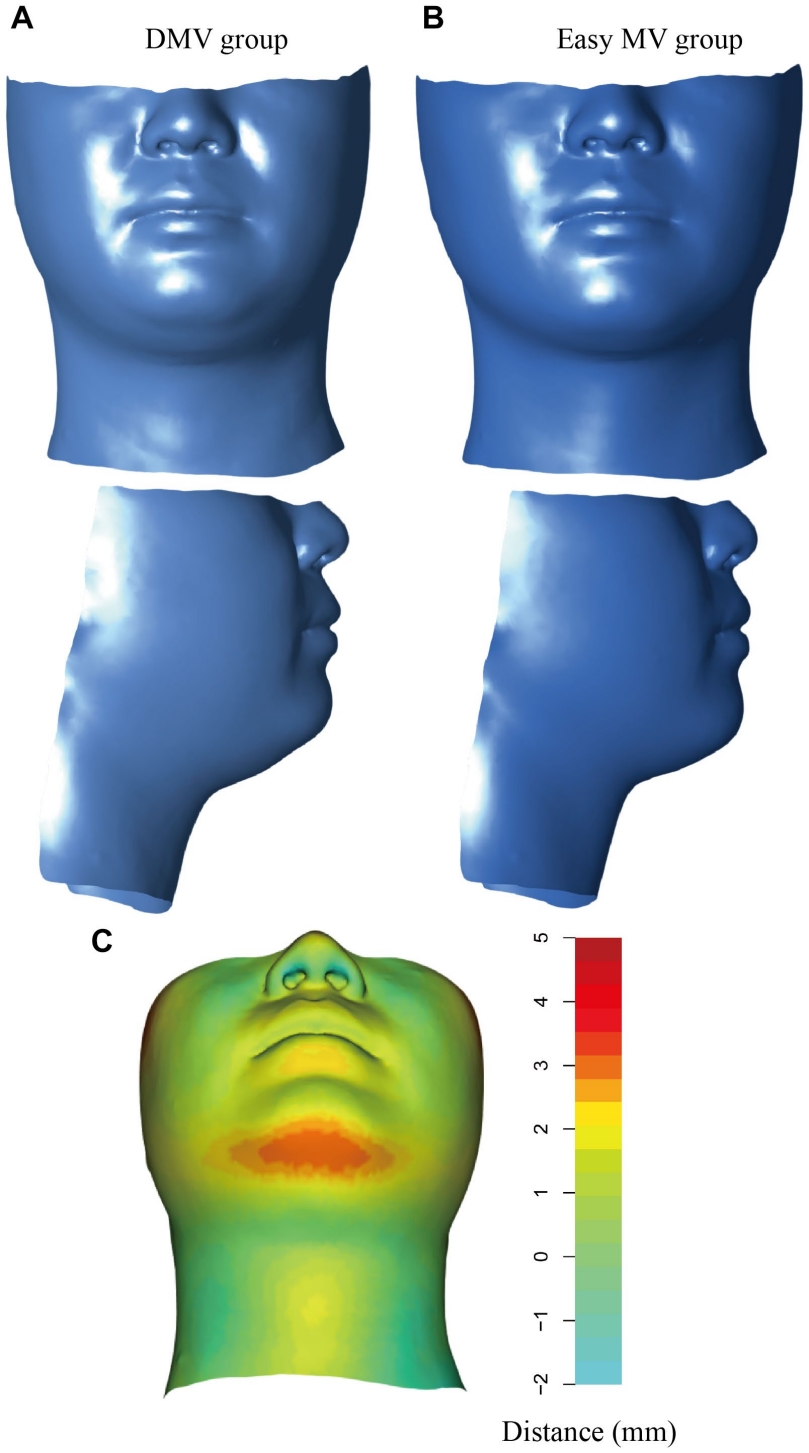
Visualization of the DMV group and the easy MV group. **(A)** Mean shape for the DMV group. **(B)** Mean shape for the easy MV group. **(C)** Colors represent the distances from the mean shape of the DMV group and the mean shape of the easy MV group. DMV, difficult mask ventilation; MV, mask ventilation.

The average shape was computed based on all the sample shape vectors in the DMV group and easy MV group (Figures 5A,B). The differences in shape between the DMV group and the easy MV group was shown in Figure 5C. The most obvious difference between the two groups could be observed in the mandibular region.

## 4. Discussion

This study aimed to demonstrate the association between maxillofacial geometry and the risk of DMV while developing a prediction model for DMV with morphometric data and ML algorithms. Our study suggested that using only the first 3 PCs as inputs, with the LR algorithm allowed for effective DMV prediction, achieving an AUC of 0.825 (95% CI, 0.765–0.885), which outperformed the DIFFMASK score.

During the preliminary test, the model exhibited its best performance with only the first 3 PCs. However, as the number of PCs increased, the overall trend was a decline in performance. This suggests that the first 3 PCs were sufficient in capturing the essential characteristics of the 3D morphological data. After 14 PCs, the performance of the model continued to decline which can be attributed to the curse of dimensionality commonly seen in morphometric data-based classification tasks (25). The later PCs might capture noise rather than meaningful information, thereby increasing data complexity and necessitating larger sample sizes.

Based on the results obtained from the preliminary test, when modeling with the first 2–5 PCs and the first 13–15 PCs, the best-performing model among the 10 ML algorithms tested was achieved by using the first 3 PCs with LR. LR is commonly employed as a modeling approach for binary outcomes in epidemiology and medicine (26). Despite the growing popularity of more complex ML algorithms, LR consistently demonstrated comparable performance and, in some cases, can even outperform these complex ML algorithms (27, 28). Across different ML algorithms in clinical risk prediction, there was considerable variability, whereas LR was generally regarded as stable (29). Complex ML algorithms such as ANN and SVM have the advantage in capturing nonlinear relationships in the data, but our data might not have exhibited strong nonlinear patterns. Furthermore, complex ML algorithms are most suitable for medical prediction problems with large datasets, whereas LR modeling requires less data and is particularly advantageous when working with relatively small datasets (30).

The human face contains a wealth of pathophysiological information, numerous studies have investigated the relationship between facial images and diseases such as coronary artery disease (31) and acromegaly (32). In the field of anesthesia, facial images have been developed to classify intubation difficulty which showed a good performance with an AUC of 0.864 (6). Although 2D image acquisition is straightforward, it is more susceptible to variations such as camera angle, focal depth, and lighting. Counterintuitively, 2D images are more complicated than 3D meshes due to their high dimensional and intricate color image variation that is nonlinear. Consequently, processing 2D data requires the use of large, complex, nonlinear network architectures and substantial training datasets. Conversely, the distribution of 3D meshes can be efficiently approximated by multivariate Gaussian distributions and analyzed using geometric morphometrics (33). With the development of 3D devices, the potential of 3D scans for predicting disease has been validated. For example, 3D facial morphology has been introduced in the discrimination of genetic syndromes such as 22q11 deletion syndromes and fetal alcohol syndrome (34, 35). More recently, 3D craniofacial scans have been developed to build the prediction model of OSAS with an AUC of 0.70 and a sensitivity of 74% (9).

Our study exemplified the application of 3D scans to DMV predicition. Mask ventilation is a fundamental technique used in general anesthesia. Currently, the prediction of DMV relies mainly on patient history and traditional bedside examinations (36). A prospective study of 1,502 patients identified five risk factors to be significantly associated with DMV including age > 55 years, BMI > 26 kg/m^2^, lack of teeth, history of snoring, and presence of a beard (20). Similarly, our study found that age, BMI and history of snoring showed significant differences among DMV and easy MV group. However, the diagnostic accuracy of DMV prediction based on these factors has been proven to be poor, with up to 94% of DMV patients ultimately failing to be predicted (37). For this reason, the DIFFMASK score (which incorporated age, sex, BMI, history of difficult intubation, history of snoring, thyromental distance, Modified Mallampati test, beard, sleep apnea, and history of neck radiation) ranging from 0 to 18 points was developed and validated in a large cohort of 46,804 patients (11). Patients with a sum score ≥ 5 were deemed to be at risk for DMV. Our study validated the predictive value of this score, with an AUC of 0.785, and different from the previous study, the optimal cut-off value was 4. This might be attributed to the absence of patients with a beard and relatively few patients with a history of neck radiation and sleep apnea. In our study, the LR model with morphometric data outperformed the DIFFMASK score. This may potentially be explained by the extensive range of information carried by facial morphology, including age (38), gender (39), and most notably, the distribution of soft tissue across the region of the face and neck, which cannot be described through BMI.

We computed the average shapes of the DMV group and easy MV group, it was apparent that the DMV group exhibited excessive soft tissue in the mandibular region, which potentially altered compliance of the upper airway wall and narrowed the upper airway lumen, resulting in airway collapse during anesthesia.

To our knowledge, no prior studies have explored the relationship between facial anatomy and DMV. However, several studies have identified specific craniofacial features in patients with difficult intubation (DI). There was a relationship between DMV and the incidence of DI. The past study verified that patients with DMV experienced a higher incidence of DI compared to those with easy MV (20). A study conducted among Japanese reported that patients who had difficulty with intubation had an increased submandible angle, which is formed by the intersection of the line between the tragus and the mentum with the submandible line (40). Another study conducted on 80 Caucasian males revealed that individuals with DI had a significantly greater jaw-neck slope compared to those with easy intubation (41). Similarly, our study confirmed that patients with DMV had such maxillofacial structures. These morphological differences can partially explain the association between DMV and DI.

The incidence of DMV varies among reported studies, possibly due to the absence of standard criteria for its definition. The ASA Task Force’s definition was subjective and vague (42) while Han et al.’s was considered too stringent and potentially led to an underestimation of DMV incidence (43). Therefore, the definition by Langeron et al. (20) was utilized in this study. It is important to note that different definitions of DMV may result in variations in incidence and can potentially impact the performance of predictive models.

There were still some limitations in this study. Firstly, the sample was limited to Chinese Han adults and may not be generalizable to other ethnic groups or younger populations. Given that facial morphology differs across races and age groups, further investigations including diverse populations are warranted to determine the association between facial features and DMV. Secondly, the study exclusively focused on patients scheduled for elective surgery who were able to undergo a 3D scan while awake and cooperative. Consequently, the model developed may not be applicable to critically ill patients or emergency surgical scenarios. Lastly, it is important to note that further research is needed to validate the prediction model’s performance on various 3D scanning devices, including handheld ones, to support its use in clinical practice.

In conclusion, this was the first study to use 3D facial scans combined with a machine learning algorithm (here is LR) to build the prediction model for DMV which achieved a good performance. The visualization demonstrated the shape differences between DMV and the easy MV group. This non-invasive and convenient approach has promising applications for DMV prediction. Nevertheless, further studies are required to validate the generalizability and clinical utility of this novel tool on a larger scale.

## Data availability statement

The raw data supporting the conclusions of this article will be made available by the authors, without undue reservation.

## Ethics statement

The studies involving humans were approved by the Ethics Committee of Shanghai Ninth People’s Hospital (no. SH9H-2020-T233-1). The studies were conducted in accordance with the local legislation and institutional requirements. The participants provided their written informed consent to participate in this study.

## Author contributions

MX and HJ contributed to the conception of the study. BP, CJ, SC, and MX contributed to the methodology of the study. BP and CJ contributed to the collection and assembly of data. BP and NJ contributed to the data analysis and interpretation. BP, CJ, MX, and HJ contributed to the writing, review, and editing of the manuscript. All authors contributed to the article and approved the submitted version.

## Funding

This study was supported by the Clinical Research Program of Ninth People’s Hospital, Shanghai Jiao Tong University School of Medicine (no. JYLJ202013).

## Conflict of interest

The authors declare that the research was conducted in the absence of any commercial or financial relationships that could be construed as a potential conflict of interest.

## Publisher’s note

All claims expressed in this article are solely those of the authors and do not necessarily represent those of their affiliated organizations, or those of the publisher, the editors and the reviewers. Any product that may be evaluated in this article, or claim that may be made by its manufacturer, is not guaranteed or endorsed by the publisher.

## Supplementary material

The Supplementary material for this article can be found online at: https://www.frontiersin.org/articles/10.3389/fmed.2023.1203023/full#supplementary-material

## Footnotes

^1^ https://meshmixer.com/

^2^ https://www.slicer.org/

^5^ https://cran.r-project.org/bin/windows/base/
