# Supplementary material for: Geometric morphometrics and machine learning from three-dimensional facial scans for difficult mask ventilation prediction
Source: Front Med (Lausanne). 2023 Aug 10;10:1203023. doi: 10.3389/fmed.2023.1203023 (PMC10447910; doi:10.3389/fmed.2023.1203023)
Supplement: Supplementary file 1 [file Table_1.DOCX]

**Table S1 The AUC (95% CI) of the models with 3 different reference meshes using LDA evaluated by LOOCV**

|  | AUC (3 PCs as input) | *P* value |
| --- | --- | --- |
| Reference mesh 1 | 0.811 (95% CI, 0.754-0.868) | 0.444 |
| Reference mesh 2 | 0.806  (95% CI, 0.744-0.869) | 0.212 |
| Reference mesh 3 | 0.808  (95% CI, 0.749-0.866) | 0.417 |

AUC, the area under the receiver operating characteristic curves; CI, confidence interval; LDA, linear discriminant analyses; LOOCV, leave-one-out cross-validation; PCs, principal components
